# The effect of Soteria on personal recovery in early episode psychosis - a two-year naturalistic cohort comparison with care as usual

**DOI:** 10.1007/s00127-026-03081-9

**Published:** 2026-04-13

**Authors:** Pien Leendertse, David van den Berg, Stynke Castelein, Cornelis Lambert Mulder

**Affiliations:** 1 GGZ Westelijk Noord Brabant, Institute for Mental Healthcare, Postbus 371, 4600 AJ Bergen Op Zoom, Netherlands; 2 Department of Psychiatry, Erasmus University Medical Center, Rotterdam, Netherlands; 3 Department of Clinical Psychology, VU University and Amsterdam Public Health Research Institute, Amsterdam, Netherlands; 4 Research and Innovation, Parnassia Psychiatric Institute, The Hague, Netherlands; 5 Lentis Research, Lentis Psychiatric Institute, Groningen, Netherlands; 6 Faculty of Behavioral and Social Sciences, Clinical Psychology and Experimental Psychopathology, University of Groningen, Groningen, Netherlands; 7 Antes, Institute for Mental Healthcare, Parnassia Psychiatric Institute, Rotterdam, Netherlands

**Keywords:** Soteria, Personal recovery, Psychosis

## Abstract

**Background:**

Soteria houses are small-scaled residential settings for the inpatient treatment of acute, early episode psychosis, in which recovery is pursued by offering a calming, normalizing environment and being present. The current study aims to compare the longitudinal effect of Soteria on Personal Recovery (PR) with community-based care as usual (CAU) in the Netherlands.

**Methods:**

The two year course of PR both on individual level and mean group level was followed in patients receiving Soteria (N = 28) or CAU (N = 94). PR was assessed with the I.ROC. As potential confounders baseline scores of psychosis symptom severity (PANSS-R), impairment in functioning (WHODAS), internalized stigma (ISMI), and hospital admissions were assessed. Individual change scores and multilevel analyses were used for comparing the course of I.ROC scores between Soteria and CAU.

**Results:**

I.ROC scores in the Soteria condition improved more than in CAU, both on individual and mean group level. Multilevel analysis revealed 3 points higher I.ROC scores for the Soteria group after two years, however the effect became non-significant after correcting for baseline measures of symptom severity and impairment in functioning.

**Conclusions:**

Although concepts like Soteria show promise to transform inpatient services for acute early psychosis in a recovery-oriented manner, we were unable to demonstrate robust effects of Soteria compared to CAU after 2 years, when accounting for between‐group differences. Potential explanations and recommendations for future research are discussed, illustrating the need for more research into ways to promote PR from early episode psychosis in inpatient care.

## Introduction

Soteria is an example of a small-scale facility for the inpatient treatment of acute, early episode psychosis, aiming to facilitate recovery by focusing primarily on interpersonal factors. Recovery from psychosis is pursued by offering a warm, homelike, normalizing environment, with being present as the most important therapeutic ingredient [1]. Soteria was developed as a research project by the American psychiatrist Loren Mosher in the 70s, aiming to find an alternative approach to recovery from psychosis than the dominantly biological treatment in the general hospitals [2]. Mosher emphasized a phenomenological approach, in which understanding the story behind the psychosis, without judging or interfering, was important for patients to be able to integrate the psychotic crisis into their lives. Fitting in the time of antipsychiatry, the primary aim was to demonstrate that recovery from psychosis without or with reduced dosages of medication was possible. Although the research project ended, the Soteria paradigm remained. Over the years, the focus of Soteria shifted from demonstrating the possibility of medication free treatment, towards a recovery-oriented treatment of psychosis by offering a calming, tension reducing environment, in which interventions on biological (including low dose medication), social and psychological level can be integrated [1]. The research into Soteria has been reviewed and consisted of three controlled trials involving a total of 223 participants diagnosed with first- or second-episode psychosis [3]. Compared to inpatient care as usual (CAU) Soteria yielded equal results in reducing symptomatology and improving functioning, although in one comparative prospective study Soteria resulted in a better work and housing situation at two year follow up [4]. However, there is a need for more extensive quantitative research comparing Soteria to CAU [3].

The emphasis on interpersonal factors in the pursuit of recovery in Soteria – next to symptomatic and functional improvement—suits the descriptions of personal recovery (PR). Personal recovery can be regarded as the patient-based definition of recovery, capturing processes like connectedness, hope, identity and overcoming stigma, meaning in life and empowerment [5]. PR is a highly personal process, and self-report measures have been developed aiming at assessing PR [6]. Personal, clinical, and functional recovery represent related yet distinct constructs, each characterised by its own developmental trajectory [7]. Using PR as an outcome measure is a relatively recent development, and therefore not included in previous quantitative Soteria-research that was performed in the 70s to 90s.

Several qualitative studies into the working mechanisms of Soteria have been undertaken across different countries [8–10]. The current study was undertaken in a Soteria house in the Netherlands. Previous qualitative research into this specific house suggested that the experience of togetherness in contact with peers and staff was one of the most important facilitators of PR from psychosis [10]. Patients emphasized the normalizing nature of contact and context, implying that the effect of Soteria might be in part due to the reduction of internalized stigma. In contrast, regular hospital admissions are sometimes mentioned by service users as stigmatizing, and stigma is one of the important barriers in reaching PR from psychosis [11].

The aim of the current study is to compare early episode patients from Soteria with Care as Usual (CAU) for its quantitative effects on PR during a 2-year follow-up period. We controlled for the potentially confounding effect of psychosis symptom severity, functioning, internalized stigma, and previous hospitalizations, based on the outcome of previous research into Soteria [3].

## Method

### Design

A two-year naturalistic cohort comparison of the effect of Soteria versus CAU on PR in the treatment of patients with early episode psychosis.

### Participants

Both Soteria patients and the CAU group participated in a multi-center longitudinal cohort study into determinants of recovery in psychotic disorders named UP’S: A Cohort Study on Recovery in Psychotic Disorder Patients [12]. Patients of ten mental healthcare institutions in the Southwest of the Netherlands will be followed over the course of ten years. Measures of clinical, functional, social and personal recovery and potentially relevant determinants will be collected yearly. For the current study, we used data from the baseline (T0), one year (T1) and two years (T2) follow up of UP’S. The inclusion period ran from 2017 to 2023. Included were patients with a primary diagnosis of a psychotic disorder according to DSM-5, aged between 18 and 65 years old and treated in an outpatient facility. Soteria had the same inclusion criteria, however, patients were aged between 18 and 35 years old and admitted to Soteria during baseline assessment. For the sake of generalizability, only participants of the CAU group aged 35 years or younger at the time of inclusion were included in this study. Excluded were patients with insufficient command of the Dutch language. The UP’S study was approved by the Regional Committee for Medical and Health Research Ethics in Rotterdam (Registration code: MEC-2017–340). See [12] for an extensive description of the study protocol.

### Assessments

At every participating location, eligible participants were selected randomly through a search in the electronic patient files of all patients fulfilling the inclusion criteria, to ensure random inclusion. After evaluation of the clinician involved, student researchers (psychology or medicine master students) approached patients, gave information about the study, and asked for informed consent to participate. The student researchers participated in the teams and received training and supervision in the assessment, scoring and entering of the data. Follow up assessment was also done by student researchers.

### Interventions

#### Soteria group

Soteria patients were admitted to Soteria between 2017 and 2020. Due to the closure of Soteria (2021, The Netherlands), the inclusion period was shorter here than at the other study sites of UP’S. Patients were in need of hospitalization because of a psychotic episode and were referred to Soteria either directly from the emergency service, from a (closed) general admission wards, or from a flexible assertive community treatment team (FACT). Assignment to Soteria was based on availability, if there was no capacity at Soteria, patients were referred to a general admission ward. The duration of stay in Soteria ranged from several weeks to several months, with an average duration of stay of two months. At follow-up, patients were either treated by FACT teams, or no longer in psychiatric care.

#### Treatment setting of Soteria

Treatment according to the Soteria model in this specific Soteria facility took place in a residential building – able to accommodate ten patients—located at the edge of the psychiatric campus. The staff was instructed to offer continual personalized ‘being with’ the patients. The home-like environment, including running a shared household between patients and staff, served as holding for symptoms of disorganization. Conceptual continuity, meaning a stable and supporting relationship between patients and staff during the whole process, from the acute phase to rehabilitation, was realized by a small team working in 12-h shifts. Besides nurses, the multidisciplinary team consisted of experts by experience, a hostess, social worker, psychiatrist and psychologist. All members of the team were approachable and visible. The group approach emphasized peer-support and offered normalizing contacts aimed at reducing self-stigma. Either no medication or consensual low dose antipsychotic strategies, in accordance with guidelines, were offered, accompanied by evidence based psychotherapeutic interventions like cognitive behavioral therapy (CBT), trauma-focused therapy and systemic therapy. Treatment took place in close collaboration with family members and other people of reference, and members of the FACT teams. Clear and concordant information about psychosis was offered on request to both patients, family and other parties involved. A structured day-program ran five days a week, and was organized outside of the mental healthcare system in collaboration with local entrepreneurs. In this program group conversations were alternated with activities focusing on physical well-being (fitness, yoga, equi-therapy) and creative expression to work on themes like identity, hope and meaning (music-groups, art-therapy). The final phase of treatment was dedicated to rehabilitation goals and a smooth transition from in- to outpatient treatment. This was done in close collaboration with the FACT team, and people of reference. Gradually the program was replaced by personal goals, spending more time at home and resume work or school.

#### Care as usual group

For the CAU group we selected all patients aged 35 years or younger, who were included at one of the other participating study sites of UP’S, and received outpatient treatment either in specialized early intervention services, or FACT teams. Treatment was offered according to the Dutch care standard for psychosis (AKWA) [13] and consisted of multidisciplinary outreaching care (the composition varied between teams but typically consisted of nurses, psychiatrists, psychologists, social workers and peer workers). Attention is paid to both medical-psychiatric care as to psychosocial treatment of psychosis. Comparable to Soteria, treatment as usual typically comprised pharmacotherapy, cognitive-behavioural therapy, nursing care, and family or systemic interventions, and—when indicated—social work and vocational support, and peer-facilitated group interventions. However, in contrast to Soteria’s emphasis on personalized “being with” and the provision of a calming, normalizing milieu, these teams primarily focus on symptom reduction. Furthermore, the frequency of contact is tailored to clinical needs, ranging from weekly to bimonthly, with the possibility of daily contact during acute phases. In case of need of hospitalization during follow-up, patients were admitted at the local general admission ward. At the time of follow-up patients were treated either in the same treatment team, or were no longer in psychiatric care.

### Instruments

#### Personal recovery

PR was assessed with the Individual Recovery Outcomes Counter (I.ROC). The I.ROC is a 12-item self-reported measurement tool that was created to measure well-being and the recovery process. It is an easy to use and preferred questionnaire for assessing recovery in clinical practice [14], it is developed in collaboration with service users and grounded in the HOPE framework (home, opportunity, people, and empowerment). Conceptually, the HOPE framework aligns closely with the CHIME model [5], supporting the use of the I.ROC as a recovery-oriented outcome measure within contemporary theoretical frameworks.

Each of the HOPE subscales comprises three questions about how it has been in the last three months; these questions are answered on a six-point scale ranging from “never” (1) to “always” (6), scores range from 12 to 72. Higher scores indicate better PR. Cronbach’s α for the current sample showed good internal consistency, based on a one-factor solution (McDonald’s Omega = 0.838). Since not all patients completed the study and others skipped a measuring moment, there are patients with one measure (T0 only), two measures (T0 and either T1 or T2) and three measures (T0, T1 and T2). We used endpoints after one year or, when available, two years.

#### Clinical characteristics

Psychosis symptoms were assessed with the Positive And Negative Syndrome Scale-Remission Tool (PANSS-R) [15]. For this short version of the PANSS eight interviewer-rated items are scored from absent (1) to extreme (7). Higher scores indicate more severe symptoms. Internal consistency of the PANSS-R in a Dutch population is high (Cronbach’s α of 0.80) [16].

Impairment in functioning was assessed by The World Health Organization – Disability Assessment Schedule 2.0 (WHODAS 2.0) [16]. We used the 32-item version. All self-reported questions asses the limitations on activity and restrictions on participation experienced by an individual in the last 30 days. They are scored from 1 (None) to 5 (A lot/Unable), with higher scores referring to more limitations. Internal consistency of the WHO-DAS is high (Cronbach’s α of 0.95) [17].

Internalized stigma was assessed with the Internalized Stigma of Mental Illness scale (ISMI-10) [18]. The self-reported questions assess how much the participant agrees or disagrees with each statement, rated on a 1 (strongly disagree) to 4 (strongly agree) Likert scale with higher scores indicating more internalized stigma. The ISMI demonstrated adequate psychometric properties (Cronbach’s α of 0.83) for assessing internalized stigma in a Dutch speaking population with mental illness [19].

Previous hospital admissions in the past year were collected from the clinical files.

### Statistical analyses

Sociodemographic and clinical characteristics were summarized using means and standard deviations or percentages for categorical variables. Differences between groups were calculated using Chi-square tests and T-tests. To detect attrition bias we used baseline data comparing participants with baseline information only to participants with one or more follow up measures.

Our primary research question was investigated at both the patient and group level since these are considered as different expressions of the effect of location [20]. First, for assessing differences at an individual level, minimum important difference (MID) scores were calculated. This was done with the formula I.ROCfinal – I.ROCbaseline/S.D., where I.ROCbaseline = observed baseline I.ROC score, I.ROCfinal = observed I.ROC end score, and S.D. = baseline standard deviation. I.ROC final is the endpoint after one year or, when available, two years. Scores greater than or equal to 1, or less than or equal to − 1 were labeled “increased” or “decreased”, respectively.

Second, for assessing differences at a group level, we used multi-level (mixed) models to investigate the difference in change in PR between Soteria and CAU over the course of time. Due to the relatively small number of measurements and the fact that participants had completed only T1, only T2, or both time points—and in order to make optimal use of all available data—we used a multilevel model rather than a repeated-measures ANOVA. The latter assumes equal numbers of observations and equal intervals, which in our case would have forced us either to exclude participants or to discard measurements. In order to control for potential confounders, the following models were used: the null-model included random intercepts and effect of time, extended models included time, group (Soteria or CAU), and an interaction effect of time*group and confounding variables. Both restricted log-likelihood tests and Akaike information criterion were used for model selection.

To explore the effects of missing values we performed complete case, for both the MID and the multilevel analysis, excluding participants with missing I.ROC data on T2. IBM SPSS Statistics (Version 27) was used for all data analyses.

## Results

### Descriptions of the study sample

At the time of data analysis 320 patients had been included in the UP’S study, of which N = 122 were 35 years or younger at time of inclusion. The Soteria group consisted of 28 patients. Of these, 18 patients completed the one-year follow-up (64%), and 13 patients the two-year follow-up (46%). Some patients missed the one-year follow-up assessment, but participated in the two year follow up, consequently there were 9 (32%) patients that had only participated at baseline, 9 (32%) had two measurements (baseline and either T1 or T2), and 10 (36%) participated at all three measurements (baseline, T1 and T2). The CAU group consisted of 94 patients at baseline, 49 patients at one-year follow-up (52%), and 37 at two-year follow-up (39%). Of these n = 94, 35 (37%) only participated at baseline, 32 (34%) participated in either T1 or T2, and 27 (29%) participated in T0, T1 and T2.

Table 1 shows that on average the Soteria group was several years younger than the CAU group. Gender, mean age at which psychosis symptoms started, use of cannabis, and number of previous admissions did not differ significantly between groups. At baseline, PR scores were lower in the Soteria group as compared to the CAU group (t(120) = -2.77, p = 0.006), while the level of impairment in functioning (t(106) = 2.07, p = 0.040) and severity of psychosis symptoms (t(109) = 5.08, p < 0.005) were higher in the Soteria group compared to CAU group. The level of internalized stigma (t(115) = -0.29, p = 0.770) showed no significant difference between groups at baseline.

**Table 1:** Baseline descriptives and dropouts

Location:	Soteria	N	CAU	N	Dropout Soteria	N	Dropout CAU	N
Age (Mean, SD)	24.3 (4.78)	28	28.8 (4.57)	94	23.3 (4.64)	9	28.5 (4.09)	35
Gender (% male)	75.0%	28	69.1%	94	88.9%	9	74.3%	35
Diagnosis - Schizophrenia	3.6%	28	24.5%	94	22.2%	9	31.0%	29
- Schizofreniform disorder	25.0%		11.7%		22.2%		17.2%	
- Schizotypical disorder	21.4%		11.7%		33.3%		13.8%	
- Psychosis NOS	17.9%		33.0%		-		31.0%	
- Other	32.1%		19.1%		22.2%		6.9%	
Use of cannabis (% positive)	28.6%	28	27.7%	94	33.3%	9	22.9%	35
First psychosis symptoms (age in years: mean, SD)	20.8 (7.09)	21	20.6 (5.77)	81	23.0 (6.13)	6	21,7 (5.59)	29
Previous admissions (Median, IR)	1 (2)	25	1 (2)	94	1 (2)	7	1.5 (2)	34
I.ROC total (Mean, SD) - Home - Opportunities - People - Empowerment	47.9 (8.06) 12.3 (1.94) 12.2 (2.83) 11.7 (3.21) 11.8 (2.89)	28	52.7 (9.42) 14.1 (2.52) 13.0 (2.92) 12.3 (2.99) 13.3 (3.49)	94	47.2 (9.76) 12.6 (1.13) 12.8 (2.54) 10.9 (2.76) 11.0 (3.43)	9	52.5 (10.03) 14.4 (2.41) 12.8 (3..31) 12.4 (3.01) 13.0 (3.91)	35
PANSS total (Mean, SD)	20.4 (6.05)	25	14.1 (6.05)	86	22.3 (2.14)	7	13.6 (5.26)	30
WHO-DAS 32 (Mean, SD)	37.1 (10.33)	23	32.4 (10.51)	83	41.3 (9.35)	6	30.8 (8.98)	27
ISMI total score (Mean, SD)	19.2 (3.32)	27	19.4 (3.34)	90	19.7 (4.39)	8	19.7 (3.53)	33

PANSS (Positive and negative symptom scale – remission tool), WHO-DAS (World-health organisation dissability assessment schedule 2.0), ISMI (Internalized stigma of mental illness SCALE), I.ROC (Individual recovery outcome counter)

### Selective dropout/ attrition bias

To detect attrition bias, we compared the baseline data of patients in both groups who dropped out (baseline information only) to the data of the study completers (one or more follow-up measures). Drop-out in this context refers to attrition from the research study, not discontinuation of the treatment service. Table 1 shows that slightly more men left the study in the Soteria group. Further, in the Soteria group, there was little difference between dropouts and completers regarding age, age at first onset psychosis, use of cannabis, number or previous admissions, severity of symptoms (t(28) = -0.82, p = 0.419), internalized stigma (t(33) = 0.02, p = 0.983) and PR scores (t(35) = 0.22, p = 0.826). Level of functional impairment (t(27) = -0.91, p = 0.370) was higher in the dropouts than in the completers within the Soteria group (reducing the difference with the control group). In the CAU group, there was little difference in all variables between drop-outs and completers; PR scores (t(127) = 0.099, p = 0.921), severity of symptoms (t(111) = 0.40, p = 0.690), level of functional impairment (t(115) = 0.77, p = 0.445), and internalized stigma (t(121) = -0.44, p = 0.660).

### Effect of Soteria on personal recovery

**Fig. 1:**
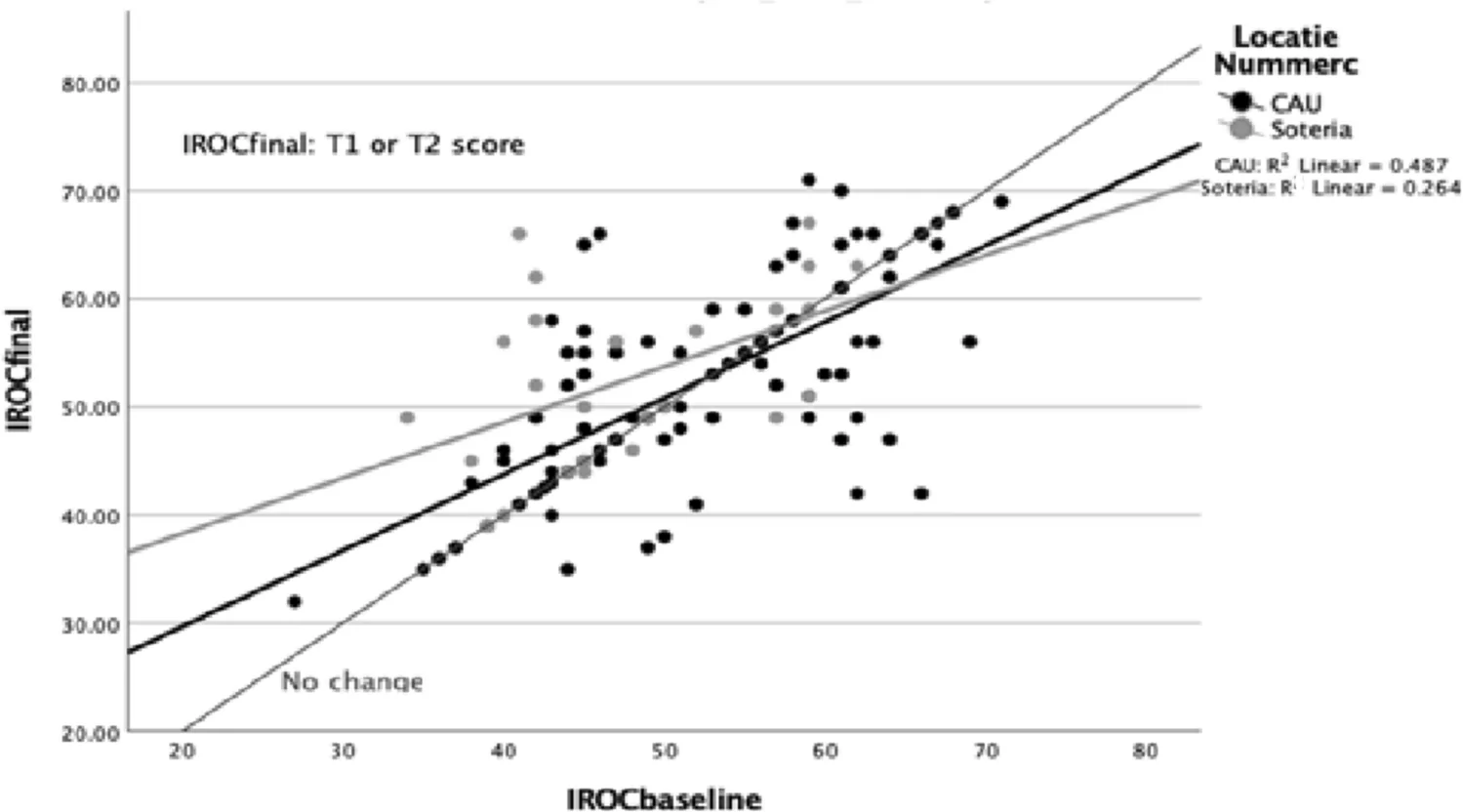
Minimum important difference of IROC for Soteria patients and CAU group

The MID of I.ROC indicated that 20% of the patients in the Soteria group, and 7% in the CAU group, showed increased I.ROC scores after 1–2 years (see Fig. 1 below; dots located left of the ´no change´ line indicate increase in I.ROC scores, dots right of the line indicate decreased I.ROC scores).

Multi-level analyses suggested that the total sample had a mean baseline PR score of 51.6, that increased 2.1 points at one-year follow-up, followed by a mean decrease of 1.3 points at two-year follow-up. Compared to the null-model (random intercept and time as fixed effect), the extended model (in which time, group and the interaction of time*group are included) suggested that although PR scores were lower in the Soteria group at baseline compared to CAU, they increased to a level equivalent to the CAU group after one year, and increased further in the second year. This resulted in a 3 points higher PR score in the Soteria group compared to the CAU group after two years (see Table 2 model 1).

**Table 2:** Results multi-level models of PR (I.ROC) by time and group (Soteria or CAU)

	***b (95%CI)***	***p***
**Model 1**		
Constant	48.55 (36.10 – 61.01)	< 0.001
Time	0.30 (0.03 – 0.57)	0.032
Group (Soteria)	4.28 (0.38 – 8.18)	0.032
Time*group	-0.31 (-0.63 – 0.00)	0.051
**Model 2**		
Constant	49.02 (45.46 – 52.57)	< 0.001
Time	0.26 (-0.02 – 0.56)	0.067
Group (Soteria)	4.56 (0.54 – 8.57)	0.027
Time*group	-0.28 (-0.61 -0.04)	0.089
Hospitalization	-2.86 (-5.92 – 0.21)	0.067
**Model 3**		
Constant	60.92 (5.93 – 115.90)	0.000
Time	0.19 (-0.06 – 0.44)	0.127
Group (Soteria)	0.74 (-3.17 – 4.64)	0.710
Time*group	-0.22 (-6.51 – 0.07)	0.136
Psychosis Symptoms	-0.59 (-0.78—-0.41)	< 0.001
**Model 4**		
Constant	63.19 (39.92 – 86.46)	0.000
Time	0.26 (0.00 – 0.53)	0.048
Group (Soteria)	2.98 (-0.80 – 6.76)	0.121
Time*group	-0.24 (-0.55 – 0.06)	0.118
Functioning	-0.41 (-0.51—-0.30)	< 0.001
**Model 5**		
Constant	73.61 (66.40 – 80.82)	< 0.001
Time	0.29 (0.05 – 0.54)	0.020
Group (Soteria)	4.55 (0.96 – 8.15)	0.013
Time*group	-0.34 (-0.62—-0.05)	0.022
Stigma	-1.30 (-1.64—-0.96)	< 0.001

Confounders are; previous hospitalization (patient chart), **baseline** symptoms severity (PANSS-R), **baseline** impairment in functioning (WHO-DAS) and** baseline** internalized stigma (ISMI**)**

Table 2 (model 2 to 5) shows the extended models in which potential confounders are included. These models suggested that baseline measurements of symptom severity, impairment in functioning and internalized stigma, were all associated with decreased PR scores. Previous hospitalization did not influence PR scores. After correction for baseline symptom severity and impairment in functioning the time*group interaction effect became non-significant.

#### Sensitivity analyses

Complete case analyses showed similar results, for both MID scores and multilevel analysis, indicating no effect of missing values.

## Discussion

### Main findings

The purpose of this naturalistic cohort comparison study was to compare PR-scores during two year follow-up of patients who had been admitted to Soteria with patients that received CAU. Overall, at the individual level, more patients in the Soteria group showed increased PR-scores compared to the CAU group, and none of the Soteria patients showed a decrease in PR at follow-up. Also, at the group level, multilevel analyses revealed better PR-scores in the Soteria group compared to the CAU group after two years. However, the effect was small and disappeared after correcting for baseline group differences in symptom severity, and impairment in functioning. Although we had a small sample with a relatively high proportion of dropouts, sensitivity analyses indicated no effect of missing data. However, at baseline, the Soteria cohort was younger and admitted for inpatient treatment, conditions that inherently increase diagnostic uncertainty. Moreover, they exhibited greater symptom severity and lower functional levels, plausibly attributable to their inpatient status. However, they may also reflect a subgroup with a more severe underlying illness trajectory compared to the CAU group. These baseline differences could have influenced the course of PR. Therefore, future studies, comparing Soteria patients to other groups of admitted patients with similar baseline psychosis symptom and functioning scores, in larger samples, are needed to investigate whether Soteria results in better PR than inpatient CAU.

### Previous research

Previous quantitative studies comparing Soteria to CAU suggested that Soteria is at least as effective as traditional inpatient services with regard to clinical and functional recovery, as summarized in the systematic review of Calton et al. [3]. This review also suggested that, because of small samples and methodological limitations, replication of findings is necessary in order to make more robust statements on the effects of Soteria. The current study aimed to add to these results by studying the effects of Soteria on changes in PR. However, although we did find small effects of Soteria on PR-scores compared to CAU, we also had to deal with small samples and are only able to make tentative conclusions about the effects of Soteria on (personal) recovery from psychosis.

Only few other studies have attempted to research the effect of interventions on PR outcomes in patients with psychosis. In a large-scale cluster RCT, outcomes of CAU plus an intervention named ‘’REFOCUS’’ were compared with those of CAU only in community mental health teams. The REFOCUS intervention [21] was a staff training designed to promote PR from psychosis through changes in the skills, knowledge, behavior, and values of staff and their relationships with service users. In this study, changes in PR did not differ between the intervention and control groups [22]. A replication of this study (REFOCUS-PULSAR) [23] was able to incorporate the (implementation) lessons learned, and their stepped-wedge cluster RCT *did* result in small but significant improvements in PR. The authors suggest this was because REFOCUS-PULSAR had people with lived experience as co-trainers [24]. Overall, these well designed, large-scaled studies illustrate the difficulty and time-consuming characteristic of significantly affecting PR in this group.

In-patient acute mental health services have been found to have difficulty addressing individuals’ needs or provide a safe and therapeutic environment. Hospitalization in early episode psychosis has been associated in literature with self-stigma and upsetting or even traumatizing experiences [25]. Previous research [26] showed that despite a significant improvement in clinical symptoms, no increase in PR scores was found before and after hospital admission in patients with psychosis. It was suggested that the process of PR might have been negatively influenced as a result of the limitations of autonomy and self-decision-making caused by coercion during hospitalisation, and the lack of training in recovery oriented care by inpatient staff [26]. In Soteria, these barriers might have been prevented, since both voluntary and involuntary patients are treated according to the recovery oriented therapeutic principles aimed primarily at PR.

Although outpatient care in specialized early intervention teams is the preferred line of treatment for early episode psychosis, outpatient care is not sufficient for all individuals in acute stages of psychosis (e.g. because of levels of risk or adverse social circumstances). For this group, which requires a specific, stigma-avoiding approach, residential acute services which offer an alternative to standard in-patient care are of considerable interest [27]. Our study might encourage more research into how to optimize user satisfaction and PR from in-patient care for psychosis.

### Limitations

Due to several important methodological limitations our results should be interpreted with caution. The first issue is the high number of dropouts in both the Soteria and the CAU group. Although the complete case analyses of both the MID and the multilevel analysis revealed no effect of missing data, our results are based on a small dataset and call for replication in larger and well-designed studies to enable us to draw firm conclusions about the effects of Soteria.

A second limitation is the difference between groups at baseline. Ideally, we would have been able to compare the Soteria group with a group of inpatient early episode psychosis patients living in the same rural area as the patients of the Soteria group. However, no such data was available. Since the Soteria group was hospitalized at baseline, their PR levels were lower and they thus had more room for improvement as compared to the CAU patients. However, the hierarchical structure of multilevel modelling helps to correct for regression to the mean. Furthermore, we controlled for several additional clinical characteristic differences between groups at baseline, including the number of previous hospitalizations (which did not appear to have an effect on PR).

Third, clinicians working within the Soteria setting explicitly endorsed to the underlying Soteria principles, whereas such affinity with, or believe in the treatment principles may be less pronounced in CAU contexts. This distinction is noteworthy, given that interpersonal factors were mentioned as one of the primary facilitating factors in reaching PR in Soteria [10]. Consequently, this potential influence cannot be ruled out as a confounding factor.Fourth, after discharge from Soteria, individuals either leave care altogether or receive follow-up treatment within a FACT team. Within the mental-health organisation in which Soteria was embedded, no specialised early psychosis team was available. As a result, specialised expertise in early-psychosis treatment becomes diluted, and the effects of treatment delivered according to the Soteria model risk being attenuated. This may have further reduced the differences in recovery trajectories between the groups.

A final limitation is the duration of follow up. We looked at the effect on PR after two years, however it might very well be that the effects of interventions are only visible after more than two years, whereby the role of time may influence how PR is perceived [28, 29]. Or, perhaps, PR as measured quantitatively, is not amendable to change by therapy, but evolves in the very long term. For example, in the total sample an increase of 2.1 points at one-year follow-up was observed in PR scores (range 12 to 72), followed by a mean decrease of 1.3 points at two-year follow-up. Since PR has a non-linear course this likely reflects normal fluctuation and measurement imprecision. However, future research should extend PR follow-up over a longer period to further elucidate this pattern and observe differences between groups.

### Clinical implications

Given the results of the present study, some elements of Soteria could be incorporated in clinical practice to make inpatient care for psychosis more recovery-oriented. Psychosis can be accompanied by experiences of depersonalization and disturbances in self-other/self-world boundaries. Recovery—among other things – might be involved with (re-)connecting to others, the self and the world [28]. Nischk & Rusch [8] suggest that the homelike environment, social context, being with and doing things together at Soteria offers relief of self-disturbances, inducing relaxation and thereby promotes (symptomatic-) recovery. In our own qualitative study of patient perspectives on facilitating and hindering factors of PR in Soteria, patients mentioned that togetherness, equal and close nature of contact, a feeling of belonging and being active in normalizing activities facilitated PR from psychosis [10]. Regular hospital admissions are sometimes mentioned by service users as stigmatizing, and stigma is one of the important barriers in reaching PR from psychosis [11]. Moreover, the climate of psychiatric inpatient units has proven to be related to outcome [30]. Even though our study design was not suitable to demonstrate an effect of Soteria on internalized stigma compared to inpatient CAU, our study hopes to inspire the pursuit of inpatient facilities incorporating elements important for PR—like enhancing connectedness, optimism about the future, increasing autonomy, and -especially—battling (self-) stigma.

### Future research

Firstly, replication of our findings on larger samples is needed to enable drawing firm conclusions about the effects of Soteria. Finding a comparable control group is needed to answer the crucial question to what extent alternatives can admit a population comparable to standard inpatient services [27]. One caveat for future research is that Soteria may be most effective for individuals who self-select into such a facility,although this self-selection might be integral to its success, it conflicts with the randomization procedures required in research.

Next, previous research based on patient-experiences with Soteria revealed that minimizing internalized stigma was one of the most important facilitators of PR [10]. Regrettably, the limited sample size precluded us from investigating the impact of Soteria on internalized stigma over time. Although groups demonstrated minimal differences in internalized stigma at baseline, including baseline measures of internalized stigma as a confounder resulted in a significant time by group interaction, which appears to underscore the relevance of stigma in the process of PR. Therefore, the effects of Soteria on internalized stigma would be an interesting topic for future research. Particularly since there is a large body of literature recognizing the role of internalized stigma in PR from psychosis (e.g. [11, 31–33]), but studies on how to decrease it are deficient. Future research should also lead to population-based normative tables for the I.ROC. To date, no peer-reviewed, normative tables have been published against which the clinical relevance of effects can be assessed.

### Conclusion

Although PR has been investigated for over a decade, it has only recently been adopted as an explicit outcome measure in psychosis interventions [34]. Intervention studies targeting PR in psychosis care remain scarce. In the current study, we aimed to gain more insight into the efficacy of Soteria as an inpatient treatment for acute psychosis. We were unable to demonstrate robust effects of Soteria on PR compared to CAU after 2 years, when accounting for between‐group differences. To conclude, existing trials [22, 24] and our study illustrate that there are still challenges of studying the effects and implementing a more recovery-oriented approach since the introduction of PR.

## Data Availability

Data are available upon request from the first author.
